# Hearing care across the life course provided in the community

**DOI:** 10.2471/BLT.18.227371

**Published:** 2019-08-20

**Authors:** Jonathan J Suen, Kaustubh Bhatnagar, Susan D Emmett, Nicole Marrone, Samantha Kleindienst Robler, De Wet Swanepoel, Aileen Wong, Carrie L Nieman

**Affiliations:** a Johns Hopkins University School of Nursing, Baltimore, United States of America (USA).; b Social Business Innovation, Medtronic Labs, New Delhi, India.; c Department of Head & Neck Surgery & Communication Sciences, Duke University School of Medicine, Durham, USA.; d Department of Speech, Language and Hearing Sciences, University of Arizona, Tucson, USA.; e Norton Sound Health Corporation, Nome, USA.; f Department of Speech-Language Pathology and Audiology, University of Pretoria, Pretoria, South Africa.; g Cochlear Center for Hearing and Public Health, Johns Hopkins University Bloomberg School of Public Health, 2024 E Monument Street, Suite 2-700, Baltimore, Maryland, 21205, USA.

## Abstract

Untreated hearing loss is recognized as a growing global health priority because of its prevalence and harmful effects on health and well-being. Until recently, little progress had been made in expanding hearing care beyond traditional clinic-based models to incorporate public health approaches that increase accessibility to and affordability of hearing care. As demonstrated in numerous countries and for many health conditions, sharing health-care tasks with community health workers (CHWs) offers advantages as a complementary approach to expand health-service delivery and improve public health. This paper explores the possibilities of task shifting to provide hearing care across the life course by reviewing several ongoing projects in a variety of settings – Bangladesh, India, South Africa and the United States of America. The selected programmes train CHWs to provide a range of hearing-care services, from childhood hearing screening to management of age-related hearing loss. We discuss lessons learnt from these examples to inform best practices for task shifting within community-delivered hearing care. Preliminary evidence supports the feasibility, acceptability and effectiveness of hearing care delivered by CHWs in these varied settings. To make further progress, community-delivered hearing care must build on established models of CHWs and ensure adequate training and supervision, delineation of the scope of practice, supportive local and national legislation, incorporation of appropriate technology and analysis of programme costs and cost–effectiveness. In view of the growing evidence, community-delivered hearing care may now be a way forward to improve hearing health equity.

## Résumé

La perte de l'acuité auditive non traitée est considérée comme une priorité sanitaire de plus en plus importante à l'échelle mondiale en raison de sa prévalence et de ses effets nocifs sur la santé et le bien-être. Jusqu'à récemment, peu de progrès avaient été accomplis pour développer les soins auditifs en dehors des modèles cliniques traditionnels de façon à intégrer des approches de santé publique permettant d'accroître l'accessibilité, notamment économique, des soins auditifs. Comme cela a été démontré dans de nombreux pays et pour de multiples problèmes de santé, transférer des tâches de soins de santé aux agents sanitaires des collectivités présente des avantages en tant qu'approche complémentaire permettant d'étendre la prestation des services de santé et d'améliorer la santé publique. Cette publication étudie les possibilités de transfert de tâches pour dispenser des soins auditifs tout au long de la vie en examinant plusieurs projets en cours à différents endroits – Bangladesh, Inde, Afrique du Sud et États-Unis d'Amérique. Les programmes sélectionnés apprennent aux agents sanitaires des collectivités à dispenser divers services de soins auditifs, du dépistage auditif chez les enfants à la gestion de la perte de l'acuité auditive liée à l'âge. Nous évoquons les leçons tirées de ces exemples pour définir les pratiques optimales concernant le transfert des tâches dans le cadre des soins auditifs dispensés dans des structures de proximité. Les observations préliminaires étayent la faisabilité, l'acceptabilité et l'efficacité des soins auditifs dispensés par les agents sanitaires des collectivités dans ces différents contextes. Pour continuer à progresser, les soins auditifs dispensés dans des structures de proximité doivent s'appuyer sur des modèles éprouvés d'agents sanitaires des collectivités. Il convient par ailleurs d'assurer une formation et une supervision adéquates, de délimiter le champ de pratique, d'adopter une législation locale et nationale favorable, d'intégrer une technologie appropriée et d'analyser les coûts du programme et le rapport coût-efficacité. Compte tenu du nombre croissant d'éléments d'appréciation, les soins auditifs dispensés dans des structures de proximité peuvent désormais constituer une solution pour améliorer l'équité en matière de santé auditive.

## Resumen

La pérdida de audición no tratada se reconoce como una prioridad sanitaria mundial cada vez mayor debido a su prevalencia y a sus efectos perjudiciales para la salud y el bienestar. Recientemente, se había avanzado poco en la expansión de la asistencia auditiva más allá de los modelos tradicionales basados en clínicas para incorporar enfoques de salud pública que aumenten la accesibilidad y asequibilidad de la asistencia auditiva. Como se ha demostrado en numerosos países y para muchas condiciones sanitarias, delegar las tareas de atención sanitaria a los trabajadores sanitarios de la comunidad (CHW) ofrece ventajas como enfoque complementario para ampliar la prestación de servicios sanitarios y mejorar la salud pública. Este documento explora las posibilidades de la delegación de funciones para ofrecer atención auditiva a lo largo de toda la vida mediante la revisión de distintos proyectos en curso en una variedad de entornos: Bangladesh, Estados Unidos de América, India y Sudáfrica. Los programas seleccionados capacitan a los CHW para que ofrezcan una amplia gama de servicios de atención auditiva, desde exámenes auditivos para la infancia hasta el tratamiento de la pérdida de audición relacionada con la edad. Discutimos las lecciones aprendidas de estos ejemplos para informar las mejores prácticas sobre la delegación de funciones dentro de la atención auditiva proporcionada en la comunidad. La evidencia preliminar apoya la factibilidad, aceptabilidad y efectividad de la atención auditiva proporcionada por los CHW en estos variados entornos. Para seguir avanzando, la atención auditiva proporcionada en la comunidad debe basarse en modelos establecidos de los CHW y garantizar una formación y supervisión adecuadas, la delimitación del campo de aplicación, el apoyo de la legislación local y nacional, la incorporación de la tecnología adecuada y el análisis de los costes de los programas y la relación coste-eficacia. En vista de las pruebas cada vez más numerosas, la atención auditiva proporcionada en la comunidad puede ser ahora una solución para mejorar la equidad en la salud auditiva.

## ملخص

يُعتبر فقدان السمع غير الخاضع للعلاج كأولوية صحية متزايدة على مستوى العالم وذلك بسبب انتشاره وآثاره الضارة على الصحة والرفاهية. وحتى وقت قريب، تم إحراز تقدم ضئيل في توسيع نطاق رعاية السمع بما يتجاوز النماذج الإكلينيكية التقليدية لدمج مناهج الصحة العامة التي تزيد من إمكانية الوصول إلى رعاية السمع والقدرة على تحمل تكلفتها. كما هو واضح في العديد من الدول وبالنسبة للعديد من الظروف الصحية، فإن تحويل مهام الرعاية الصحية إلى العاملين في القطاع الصحي المجتمعي (CHWs)، يوفر المزايا كنهج تكميلي لتوسيع نطاق تقديم الخدمات الصحية وتحسينها. تستكشف هذه الورقة قدرات تحويل المهام على توفير رعاية السمع عبر دورة الحياة من خلال مراجعة العديد من المشاريع الجارية في مجموعة متنوعة من الأوضاع – بنغلاديش والهند وجنوب أفريقيا والولايات المتحدة الأمريكية. تقوم البرامج المختارة بتدريب العاملين في القطاع الصحي على توفير مجموعة خدمات رعاية السمع من مرحلة الفحص سمع في الطفولة إلى التعامل مع فقدان السمع المرتبط بالتقدم في السن. نحن نناقش الدروس المستفادة من هذه الأمثلة للاطلاع على أفضل الممارسات لتحويل المهام في قطاع خدمات رعاية السمع المقدمة من المجتمع. تدعم الأدلة الأولية مدى جدوى رعاية السمع المقدمة من العاملين في القطاع الصحي، ومدى مقبوليتها وفعاليتها في هذه الأوضاع المختلفة. لتحقيق مزيد من التقدم، يجب أن تعتمد رعاية السمع المقدمة في المجتمع على نماذج راسخة من العاملين في القطاع الصحي، وضمان التدريب والإشراف الكافيين، وتحديد نطاق الممارسة والتشريعات المحلية والوطنية الداعمة، ودمج التكنولوجيا والتحليل المناسبين لتكاليف البرنامج وفعالية التكلفة. في ضوء الأدلة المتزايدة، قد تكون رعاية السمع التي يقدمها المجتمع الآن الطريق إلى الأمام لتحسين انتشار الصحة السمعية

## 摘要

因其普遍存在且对健康和福祉有不利影响，未及时治疗的听力损失仍被视为日益增长的全球健康优先事项。直至近期，在将听力保健扩展到以诊所为基础的传统医疗模式之外，并将其纳入公共卫生渠道、提高听力保健的可获得性和可负担性方面，几乎没什么进展。正如诸多国家的健康状况所表明的那样，将卫生保健工作移转给社区卫生工作者可以带来便利，同时可作为扩大卫生服务提供范围和改善公共医疗的一种补充方法。本文通过回顾孟加拉国、美利坚合众国、南非和印度等国不同环境中的几个正在进行中的项目，探讨了工作移转在提供终身听力保健方面的可能性。所选课程能培训社区卫生工作者提供一系列听力保健服务，从儿童听力筛检到与年龄有关的听力损失治疗。我们讨论了从这些例子中汲取的经验教训，以在社区范围内提供的听力保健工作移转中实施最佳实践。已有初步证据支持社区卫生工作者在这些不同环境下提供听力保健的可行性、可接受性和有效性。为了取得进一步进展，社区范围内提供的听力保健必须建立在社区卫生工作者的现有模式基础上，并确保充足培训和适当监督、划定实践范围、支持地方和国家立法、纳入适用技术以及项目成本-效益分析。越来越多的事实表明，如今，社区范围内提供的听力保健或可能成为改善听力健康公平性的一种途径。

## Резюме

Нелеченная потеря слуха получает все больший глобальный приоритет здравоохранения в связи с ее распространенностью и негативным влиянием на здоровье и самочувствие. До последнего времени отмечен лишь незначительный прогресс в распространении охраны здоровья уха и слуха, помимо основанных на клиническом лечении традиционных моделей для включения общественных подходов к охране здоровья уха и слуха, повышающих его доступность и осуществимость. Как показано во многих странах и для многих состояний здоровья, перенос задач здравоохранения на общинных медико-санитарных работников (ОМСР) обладает рядом преимуществ, таких как вспомогательный подход для расширения доступа к здравоохранению и улучшения общественного здоровья. В данной публикации изучаются возможности переноса задач обеспечения здравоохранения в течение всей жизни путем анализа нескольких продолжающихся проектов в различных условиях (Бангладеш, Индия, Соединенные Штаты Америки и Южная Африка). Отобранные программы направлены на обучение ОМСР предоставлению ряда услуг охраны здоровья уха и слуха: от скрининга слуха у детей до возрастной потери слуха. Обсуждаются уроки, вынесенные из этих примеров, для информирования о наилучших практиках переноса задач в общинной охране здоровья уха и слуха. Предварительные доказательства поддерживают выполнимость, доступность и эффективность охраны здоровья уха и слуха, обеспечиваемой ОМСР в этих различных условиях. Для обеспечения дальнейшего прогресса общинная охрана здоровья уха и слуха должна строиться на установленных моделях ОМСР, обеспечивать адекватное обучение и наблюдение, ориентированность на практику, поддерживающее местное и национальное законодательство, включение соответствующей технологии и анализа стоимости и экономической обоснованности программы. В свете увеличивающегося количества доказательств общинная охрана здоровья уха и слуха может стать перспективой для улучшения обеспечения справедливости здравоохранения, связанного со слухом.

## Introduction

Fundamental to communication for many people, hearing serves as a connector throughout our lives. The loss of hearing has repercussions on individuals, families and societies. With the world’s ageing population, the condition affects a growing number of individuals, and is one of the leading causes of years lived with disability.^1^^,^^2^ An estimated 466 million people have a disabling hearing loss and this number is expected to increase to over 900 million by 2050.^3^

The global burden of hearing loss lies primarily on adults given the prevalence of age-related hearing loss. However, unaddressed hearing loss in children impairs speech and language development and literacy and educational attainment, which have direct and indirect effects on children’s health and attainment over their lifetime.^1^^,^^3^^,^^4^ Once considered a benign part of ageing, emerging evidence supports the negative impact of age-related hearing loss on almost every area of life, including cognitive, physical and psychosocial functioning.^5^

Little progress has been made in tackling the global burden of hearing loss. Few people with hearing loss use hearing aids, despite the consequences of untreated hearing loss and the existence of evidence-based treatment.^1^^,^^5^ Many barriers exist to greater use of hearing care, including lack of access to affordable technology, limited availability of clinic-based care, accompanying stigma and a lack of public and political awareness and prioritization of the problem.^1^^,^^5^ Calls have been made to tackle hearing loss as a public health problem and opportunities exist to draw upon public health approaches to manage hearing care at the population level.^4^^,^^6^^,^^7^ These approaches include community-based efforts that make use of local expertise to fill gaps in clinic-based care.^4^^,^^6^^,^^7^ Community-delivered models of hearing care are beginning to emerge with promising results and may be an essential approach to expand access to hearing care.^8^

## Community-delivered hearing care

Public health programmes have adapted community-delivered models to manage care within resource-constrained settings, including limited availability of professional expertise. A common solution is task shifting where community health workers (CHWs) work in partnership with professional health-care providers and under their supervision.^9^^,^^10^ The scope of practice of CHWs varies; they may conduct screenings, serve as health educators, coordinate care and provide informal counselling among other duties.^11^^–^^13^ Other related names for CHWs include *promotores de salud* (health promoters), community health aides, peer counsellors and public health aides.^9^^,^^11^^,^^14^ Here we will consider these terms as synonymous when referring to a community-based workforce. Despite the diversity of CHWs, a defining aspect of these health workers is that they typically share characteristics with their patients, such as life experiences, geography, and ethnic, cultural or linguistic traditions. Such shared qualities uniquely position CHWs to consider cultural context, environmental factors and the needs and values of their patients, and tailor services to them.^12^^,^^14^^,^^15^

CHW programmes have focused on a range of health conditions across the life course, from delivery of maternal and child health^16^ to chronic disease management.^14^ Such programmes have been implemented across the world and have been found to be effective, cost-saving and sustainable.^12^ Community-delivered hearing care programmes that employ CHWs are increasing and they are an approach that may help respond to the global prioritization of hearing care.^8^ By incorporating mobile health (mHealth) and drawing upon existing knowledge from CHW models, countries and the international community have an opportunity to answer calls to action and expand hearing care that prioritizes initiatives that aim for hearing health equity across a population.

Here, we share our experiences with several early programmes in Bangladesh, India, South Africa and the United States of America (USA) and lessons learnt to inform best practices in community-delivered hearing care. The programmes were selected to represent different resource-constrained settings and a range of activities, scopes and target populations, from children to older adults (Table 1). This paper considers the potential and challenges of task shifting within hearing care and is intended to complement emerging research on community-delivered hearing care.

**Table 1:** Characteristics of example community-delivered hearing care programmes

Programme	Location	Target population	CHW duties	CHWs	Training	Supervision	Support and continuing education	Financial model	Current level of evidence
**Children**									
Alaska Community Health Aide Program^17^^–^^24^	Community clinics in sites throughout Alaska, USA	School-aged children but also part of broad health-care system for both children and adults	Conduct targeted health assessments; use telemedicine technology to collect necessary information to share with specialists	Paid employees who are also members of the local community	Completion of the Alaska Community Health Aide Program (levels I–IV, practitioner)	Community health aide trainers, supervisor instructors, physicians/mid-level health professionals	Monthly teleconference meetings; required continuing education; practical requirements after completion of each training session; physician/mid-level practitioner oversight	Employees of the health-care system, providing care that is reimbursable through insurance	Standard of care for medical management in rural Alaska; ongoing multisite randomized trial for use in school screening and referral (NCT03309553)
Hearing screening of children^25^^–^^27^	Early childhood development centres in sites around South Africa	Low-income preschool children	Conduct hearing screening; raise awareness of early childhood development teachers	Paid lay community members	Informal training on hearing health awareness and hearing screening	Remote internet-based monitoring of screening quality by programme manager	Retraining based on quality indicators (mhealth monitoring); monthly meetings to discuss programme	Employed through grant funding	Pilot studies completed; ongoing programme with systematic evaluation underway
**Adults and older adults**									
*Oyendo Bien*^28^^,^^29^	Housing complexes and community meeting spaces for older adults in Santa Cruz County, Arizona, USA	Rural and low-income older Spanish-speaking adults	Recruit clients and raise awareness; facilitate five-week group programme on hearing health education and training on communication strategies	Paid employees (*promotores de salud*) of a public community health centre	Prior work experience in health education; three-hour training on hearing; 24-hour multisession training for facilitators	Collaborating audiologists	Weekly meetings; three-hour continuing education training at one year	Employed with health centre; work hours contracted through grant funding	Pilot studies completed; ongoing randomized controlled clinical trial (NCT03255161)
*Shruti*^30^^,^^31^	Low-income neighbourhoods in sites around Bangladesh and India	Low-income children, adults and older adults in urban and rural settings	Conduct door-to-door screening and organized screening events using smartphone technology; fit low-cost digital hearing aids	Paid employees of health-care providers	Informal training on hearing health and use of hearing screening, technology	Remote internet-based transmission of patient information to supervising ear, nose and throat surgeon	Weekly meetings of operations team with ear, nose and throat surgeon; bi-annual refresher training	Employed by ear, nose and throat surgeon; revenue from device sales off-set costs related to technology and staff	United Nations case study; ongoing programme with systematic evaluation
HEARS^29^^,^^32^	Housing complexes and community centres for older adults in Baltimore, USA	Low-income older adults	Deliver two-hour programme to older adults with individual aural rehabilitation; provide, fit, and familiarize older adult with hearing device	Peer educator – active older adult embedded in housing communities for older adults	Eight 90-minute didactic classroom modules with one practical session for certification	Collaborating audiologist and trainers	Monthly telephone calls for case reviews; monthly two-hour in-person continuing education group sessions	Project funded by research grant with volunteer peer educators; potential for reimbursable care through insurance	Pilot studies completed; ongoing multisite randomized controlled clinical trial (NCT03442296)

CHW: community health worker; mhealth: mobile health technology.

## Innovative models: children

### Remote telemedicine care

Remote communities are at a higher risk of untreated hearing loss because of the scarcity of specialists and the long distance to reach health-care services. To overcome these barriers, policy-makers in Alaska, USA, formally introduced a community-based model in 1968 in which community health aides deliver frontline care in more than 250 village clinics.^17^^,^^18^ Community health aides are trained in basic primary and emergency care, including ear and hearing care. These aides are supported by specialists through a state-wide telemedicine network.^19^^,^^20^ In the case of an ear or hearing problem, community health aides take an otological history, and perform an examination and hearing screening. The results are then transmitted to an audiologist for review and referral to an ear, nose and throat surgeon as needed. Clinicians give recommendations to the health aide, which varies from prescribing local management in the village clinic to making an appointment for further evaluation at a field clinic or travel to the tertiary care hospital for surgery. Through this workforce of community health aides embedded within a telemedicine infrastructure, the programme provides more than 4000 ear and hearing consultations a year.^21^^–^^23^

The community health aide model, with specialty triage provided through a telemedicine network, has been used for clinical care for more than 15 years, but has only recently been used for a population-based preventive screening programme for hearing. A randomized trial is being conducted in 15 communities throughout north-west Alaska that uses mHealth smartphone-based hearing screenings, and referrals through the telemedicine network to improve the effectiveness of the school-based hearing screening and referral processes in remote communities.^24^

The challenges of a telemedicine network supporting ear and hearing care delivered by community health aides include training and equipment maintenance. Regular and frequent training of community health aides is necessary to ensure quality and encourage continued use of mHealth technologies. Telemedicine equipment, such as video otoscopy and audiometers, requires calibration, servicing and routine maintenance, all of which can be difficult in remote environments. Within the community health aide programme, ongoing training is provided, including continuing education and real-time training, and telemedicine equipment is actively managed at all sites.

### Mobile hearing screening

South African CHWs have historically focused on communicable diseases rather than noncommunicable conditions such as hearing loss. Research on the delivery of hearing screening by CHWs is limited, in part due to the expense of conventional equipment and the advanced training required to operate it. New mHealth technologies, such as tablet- and smartphone-based audiometers and video otoscopy, increase the range and quality of care provided by CHWs.^33^ Validated mHealth technologies^34^ allow hearing screening to be done in primary care clinics^35^^,^^36^ using automated testing and a smartphone interface. These technologies enable personnel to provide reliable remote hearing screening for high-risk children.^25^^–^^27^ Beginning in the early 2000s, South African CHWs, using mHealth technologies, have also delivered hearing screening to children in a variety of community settings, including home visits and early childhood development facilities.^26^^,^^27^ Evaluations of these models demonstrate that CHWs can be trained to screen children in a reliable and time-efficient manner.^26^^,^^27^ After identifying a child with hearing problems, mHealth systems allow CHWs to facilitate further intervention by notifying families and caregivers about nearby clinics for advanced hearing care.^25^ A challenge to hearing screening by CHWs is noise levels that influence referral rates.^26^^,^^27^ While these technologies can monitor environmental noise when it becomes excessive, the screening protocol requires adjustments, especially at lower frequencies.^26^^,^^27^ Another challenge is monitoring the quality of the screening done by CHWs. To manage this challenge, the hearing screening protocol includes a randomized false presentation of a sound. If a CHW records that the person tested gave a response, which is incorrect, this record will register against the unique quality index of the health worker and can be tracked by programme managers and prompt retraining.^26^^,^^27^

## Innovative models: adults

### Culturally aligned rehabilitation

*Oyendo Bien* (hearing well) is a hearing loss education and support programme, which is designed to be culturally relevant to older Spanish-speaking adults. The goal of this group intervention is to help individuals and families manage age-related hearing loss. The programme began in 2015 and is facilitated by Spanish-speaking CHWs, who work at an Arizona community health centre on the border between the USA and Mexico. CHWs receive training in identification of the signs and symptoms of hearing loss and facilitation of a hearing-health education group.^28^

Through a collaboration involving community members and experts from audiology, public health and translation,^37^^–^^39^ the development of *Oyendo Bien* was guided by community-based participatory research principles.^29^ The main principles of *Oyendo Bien* include: participation of communication partners, who are typically family members or friends; rehabilitation services facilitated by CHWs and audiologists; training in communication strategies for individuals with hearing loss and their communication partners; and an emphasis on peer support. Preliminary results from the *Oyendo Bien* pilot programme show positive outcomes, including adoption of communication strategies by individuals with hearing loss and their communication partners, improved quality of life and increased use of hearing care by those with hearing loss.^39^

A challenge in the development of *Oyendo Bien* was a lack of training curricula for CHWs on hearing loss and rehabilitation options. The content of curricula designed for other health professionals and even the lay public, tended to be highly technical or device-centred, while the focus of *Oyendo Bien* is on rehabilitation. The training also needed to be relevant to CHWs already working in a health centre. In the end, training was co-developed with CHWs to make use of their strengths as experienced health educators.^28^ Training requirements continue to be evaluated to ensure that training remains responsive to the needs of the groups and the CHWs.

### Public–private partnership

The *Shruti* (healthy ear) programme is the result of a partnership between public and private sectors, specifically CHWs, physicians and device manufacturers, including amplification devices or hearing aids. *Shruti* is a community-based, door-to-door programme that offers hearing care for low-income people in Bangladesh and India.^30^ The programme is delivered by CHWs who work with ear, nose and throat surgeons and includes the provision of a low-cost hearing aid. Through mHealth hearing screening and otoscopy technologies, CHWs document and transmit information from field assessments, including results of audiometric tests, for review by the surgeons who give their recommendations for follow-up.^30^

The programme began in 2013 and now operates in 65 hospitals and clinics. As of July 2019, more than 650 000 people have been screened. A 2017 report documented several social impacts of the *Shruti* programme, including increased awareness of and access to hearing care, improvements in work performance and social interactions in patients who had their hearing loss treated, and increased employment opportunities for CHWs.^31^

As a programme delivered by CHWs, *Shruti* depends on a team of health workers who are engaged, motivated and remain with the programme. Hiring and retention of these CHWs is a continuing challenge, which is complicated by a growing local health-care industry that offers increasing employment opportunities. Another important challenge is to optimize staff size to balance personnel costs with the delivery of high-quality services. Efforts are ongoing to make *Shruti* financially self-sustaining by scaling up the size of the programme.

### Peer educators

The HEARS (Hearing Equity through Accessible Research and Solutions) intervention is another community-delivered aural rehabilitation programme designed for older adults. The programme began in 2013 in partnership with local affordable housing organizations for older adults based in Baltimore, USA. Similar to *Oyendo Bien*, the HEARS programme includes education on hearing loss and communication strategies but, like *Shruti*, provides a low-cost, over-the-counter amplification device.^32^ The over-the-counter amplification devices used in HEARS are similar in quality to hearing aids, but are available directly to consumers in the USA.^40^ The HEARS programme capitalizes on existing resources by recruiting older active community members to partner with audiologists and who serve as CHWs to deliver interventions.

These peer educators deliver hearing care to low-income older adults with remote supervision provided by an audiologist. The HEARS model maximizes hearing care accessibility in multiple ways, including peer mentoring to help tackle mistrust of health-care professionals,^41^ theory-driven methods to enhance self-efficacy^42^ and a person-centred programme. The results of a randomized controlled pilot study showed communication improvements of a similar magnitude as earlier studies that used clinic-based care with hearing aids.^32^ Currently, the effectiveness of the programme is being investigated in a larger randomized controlled trial (NCT03442296). HEARS has been adapted for delivery to people with dementia in a memory clinic, Korean Americans in faith-based organizations and for delivery by communication disorder assistants in Canada.^43^^–^^45^

Recruiting peer educators who have the time and motivation to take on additional duties, while also often managing their own health and family obligations, is a challenge. Openness to interacting with and learning to use mobile, touchscreen technologies, such as a smartphone or tablet, can further limit the pool of potential peer educators. Furthermore, peer educators must be able to read at a basic level to complete the training, which relies on interactive and text-based instruction, although the programme does not require a certain literacy level for clients.

## Lessons learnt

Initial evidence supports the use of CHWs to deliver hearing care in a range of capacities, including screening and diagnosis of paediatric hearing loss in different settings, and the provision of aural rehabilitation and hearing technology to adults and older adults.^8^ Although not intended to be a systematic review, the programmes we reviewed illustrate common themes and challenges, and can help inform the development of preliminary best practices for community-delivered hearing care (Box 1). These best practices build on the World Health Organization’s recommendations on implementing a task-shifting approach, which was originally applied to care for people living with human immunodeficiency virus.^10^

Box 1 **Best practices in task shifting to community health workers and application to community-delivered hearing care**

**Training, supervision and continuing education**

Best practices

- Training: Competency-based, needs-driven. Based on clear procedures and protocols. Topics and teaching techniques driven by CHWs. Include full spectrum of knowledge required; skills, not limited to clinical skills alone. End in certification.
- Supervision: Structured, regular and provided by trained supervisors. Monitor with defined quality measures. Regularly assess skills. Match intensity of oversight with complexity of task.
- Continuing education: Structured, regular mentoring opportunities provided by supervisors and peers. Explicit opportunities for career advancement. Performance-based incentives. Employ CHWs whenever possible rather than relying on volunteers.

Application to hearing care

- Training: Incorporate hearing health, hearing care, relevant technology, as well as other skills, such as public speaking, teaching and empathic training, as appropriate. Ensure procedures and protocols reflect programme; cover history-taking, screening, counselling and advanced counselling, as needed.
- Supervision: By audiologists and/or ear, nose and throat surgeons, but require proficiency in supervision of CHWs. Frequency and nature of supervision (in-person or remote) reflect CHWs’ scope of practice (e.g. paediatric hearing screening or fitting older adults with amplification devices).
- Continuing education: Mentoring provided by audiologists and/or ear, nose and throat surgeons. Recognize value of peer mentoring. Create opportunities for increased responsibility.

**Scope of practice**

Best practices: Define scope of practice for each member of the care team. Foster environment of collaboration and communication. Emphasize importance of extending services, counteract threat of competition. Tailor degree of task shifting to nature of condition.

Application to hearing care: Allow scope of practice to be defined by CHWs, audiologists, ear, nose and throat surgeons, patients and other stakeholders. Encourage culture of support and open dialogue between CHWs, and audiologists/ear, nose and throat surgeons. Vary scope by condition (e.g. paediatric hearing loss versus mild/moderate age-related hearing loss).

**Local and national policies**

Best practices: Recognize importance of top-down leadership and role of policy. Involve stakeholders, including CHWs and patients, from the start. Use existing regulations or revise policies as needed. Prioritize sustainable models of care, including those that are reimbursable through insurance. Ensure policies support mechanisms for reimbursement and scope of practice. Appropriately regulate certification process for safety and quality without excess burden or exclusion of parties involved.

Application to hearing care: Ensure appropriate regulation and certification of CHWs providing ear and hearing care. For sustainability of hearing health care, work for reimbursement of telemedicine, hearing screening and counselling provided by CHWs through available avenues, such as insurance.

**Role of technology**

Best practices: Make use of technology to strengthen efficiency, monitor quality, improve performance, facilitate care, support research and evaluation, and reduce costs. Select CHWs based on technology demands of the programme. Include training on use, care and maintenance of technologies. Consider usability of technologies by CHWs and patients.

Application to hearing care: Incorporate mHealth hearing care technologies (e.g. digital otoscopy and automated hearing screening) and low-cost, high-quality amplification devices. Select CHWs with understanding of technology as appropriate. Select amplification devices based on quality and usability for patients and CHWs, not supervisors. Include training on how to use smartphones and touchscreens, as needed.

**Programme costs and cost–effectiveness**

Best practices: Track programme costs, including savings. Perform cost-analysis studies whenever possible.

Application to hearing care: Record costs of CHWs, examinations and screening technologies, including care and maintenance, and amplification devices, including over-the-counter options.

CHW: community health worker; mHealth: mobile health technology

### Training and supervision

Similar to CHWs who provide care for other noncommunicable diseases, CHWs delivering hearing care must complete competency-based training in ear and hearing care.^10^^,^^46^ This training must incorporate clear procedures and protocols that are in line with the scope of practice of the CHWs, whether related to hearing screening and referral or guiding patients through a choice of hearing technology.^10^^,^^46^ Although training must ultimately be standardized across a programme, training needs vary with the skills and experience of the individuals. Training may extend beyond ear and hearing care depending on programme demands, and may require competency in, for example, public speaking, teaching, technology (e.g. smartphones and touchscreens) and empathic training.^10^ Training of CHWs involves a range of techniques, including classroom instruction and practical learning with discussions, demonstrations and role playing.^28^^,^^47^^,^^48^

Consistent supervision, mentoring by supervisors and peers, and continuing education are critical, as is the input of CHWs so as to optimize the relevance of the topics covered and the teaching techniques.^10^^,^^47^^,^^49^ For example, the Alaskan community health aides have continuing education requirements that include practical oversight by the trainers and consultations must be signed off by a physician or mid-level health profesional.^17^^,^^18^ Within *Oyendo Bien* and HEARS, CHWs participate in refresher sessions through in-person group meetings with the audiologist trainers and booster sessions as needed. Supervisors must also consider CHW retention through fair remuneration and opportunities for career advancement, particularly in competitive labour markets.^48^

### Scope of practice

Overall, task shifting has been met with variable acceptance by health-care professionals with tension stemming from concerns around competition, as well as patient safety and quality.^46^ Maximizing the scope of practice of CHWs requires the involvement of all relevant stakeholders including CHWs and patients, a clear definition of the duties and responsibilities of each team member, and an understanding that community-delivered care is a complementary and essential model of care needed to tackle the global burden of hearing loss.^10^

Given that the most common causes of hearing loss change over the lifespan, the role of CHWs varies with the etiology of the hearing loss and associated differences in treatment. For paediatric hearing loss, CHWs in Alaska and South Africa extend the reach of existing professional hearing-care services. In contrast, for adult-onset hearing loss, particularly mild to moderate age-related hearing loss, community-delivered hearing care may shift more tasks to CHWs, who are able to deliver basic group and individual aural rehabilitation, and provide, fit and orient individuals to an amplification device. Regardless of the scope, a close working relationship between team members is needed to prevent situations that could increase the vulnerability of populations who are already at risk of exploitation.

### Policies

Government, local or national, plays a role in the success of community-delivered hearing care through regulations and reimbursement policies.^10^ In some circumstances, existing regulations may accommodate an expanded scope of practice for CHWs without legislative changes. However, a lack of regulatory support for an expanded scope of practice can severely limit the influence of hearing care delivered by CHWs.^10^^,^^46^ Formal certification, either voluntary or mandatory, can support the appropriate regulation of CHWs and their establishment as a paraprofessional workforce, protect vulnerable populations, and help ensure high-quality care.^10^^,^^47^ This certification should include background checks, patient privacy regulations, and standardization of core competencies and scope of practice. Whether through private or public insurance, reimbursement of care provided by CHWs and the use of telemedicine are variable, and reimbursement can be a key enabler of the adoption and expansion of task-shifting approaches. For example, growth of the Alaskan model of community health aides and telemedicine was driven by policy changes that allowed insurance reimbursement.^20^^,^^23^

### Role of technology

Technological advancements underpin community-delivered hearing care and include digital otoscopy, tympanometry and portable automated hearing testing. These technologies provide point-of-care diagnostics coupled with internet-based data management and low-cost, high-quality hearing technologies.^21^^,^^27^^,^^50^ These technologies allow CHWs to provide services, monitor and maintain quality, facilitate referrals based on location, collect surveillance data, and track service delivery, including follow-up care.^27^^,^^33^

With an emphasis on technology-assisted solutions, community-delivered hearing care relies on the acceptance and use of technology by CHWs, patients and their families. Selection of CHWs and training must consider the technology demands of the programme. For example, the Alaskan programme requires the community health aides to be able to use a range of devices independently and the HEARS programme requires peer educators to be open to learning how to use a smartphone to fit an amplification device. Sufficient time and resources must be dedicated to training CHWs on device use, care and maintenance. Adoption of technology by users in the long term requires them to believe that these technologies are useful, important and functional.^33^^,^^51^ Self-efficacy and ease of use of the technology influences adoption.^51^ Design and training must maximize the immediate ease of using a technology while minimizing difficulties in accessing support.^33^

### Cost–effectiveness

Data on the costs and cost–effectiveness of community-delivered hearing care are lacking.^8^ Many of the programmes described are projects that are now being evaluated in larger studies that include cost and cost–effectiveness analyses. However, evidence from other fields and disciplines shows lower costs and cost savings, and preliminary evidence also indicates cost–effectiveness.^12^^,^^52^ Models of community-delivered hearing care provided by CHWs have been identified as promising innovations to increase the affordability and accessibility of hearing care.^1^^,^^6^

## Conclusion

Although hearing loss is increasingly recognized as core to health and overall well-being, clinic-based hearing care has not adequately addressed the growing global burden. Drawing on public health approaches used with other prevalent conditions, community-delivered hearing care offers new approaches to task shifting through CHWs. These approaches expand the way in which hearing loss is diagnosed and managed across the life course. As with all global health initiatives, community and stakeholder engagement, together with evolving science and technology, and the support of government, is critical to put promising evidence into practice and improve equity in hearing health.
